# Content of nitrate and nitrite in commercial and self‐made beetroot juices and the effect of storage temperature

**DOI:** 10.1002/fsn3.3575

**Published:** 2023-07-23

**Authors:** Raul Bescos, Mark L. Rollason, Tanisha S. Davies, Patricia Casas‐Agustench

**Affiliations:** ^1^ Faculty of Health, School of Health Professions University of Plymouth Plymouth UK

**Keywords:** beet, nitrate, nitric oxide, nitrite, nitrogen

## Abstract

Popularity of beetroot juice (BJ) is growing due to its high inorganic nitrate content $\left({\mathrm{NO}}_{3}^{-}\right)$ and its potential physiological benefits. However, the content of ${\mathrm{NO}}_{3}^{-}$ is not indicated in most commercial BJs and it can be affected by seasonal changes and storage conditions. This study analyzed the content of ${\mathrm{NO}}_{3}^{-}$ and nitrite $\left({\mathrm{NO}}_{2}^{-}\right)$ in five and two commercial and self‐made BJs, respectively, that were purchased in the summer and winter periods. The effect of storage temperature (20°C, 4°C, and −20°C) and pH was also analyzed. In nonconcentrated BJs, the ${\mathrm{NO}}_{3}^{-}$ content was 34 ± 20% (*p* = .075) in the winter than in the summer. ${\mathrm{NO}}_{3}^{-}$ was fully degraded in self‐made BJ after 3 days at 20°C. This effect was attenuated by 78% and 82% when it was kept at 4°C and −20°C, respectively. The addition of lemon juice (5%) to self‐made BJ was another useful approach to avoid ${\mathrm{NO}}_{3}^{-}$ degradation for 3 days when it was kept at 20°C. Regarding ${\mathrm{NO}}_{2}^{-}$, self‐made BJ had higher concentration (0.097 ± 0.01 mg/mL) compared to commercial BJs (<0.1 mg/mL; *p* = .001). The pH of self‐made BJ was higher (6.3 ± 0.1) compared to commercial BJs (4.5 ± 0.3; *p* = .001). These results suggest that the content of ${\mathrm{NO}}_{3}^{-}$ in nonconcentrated BJs can substantially differ across the year and this is an important factor to take into account when recommending BJs to promote some of its potential physiological benefits.

## Graphical abstract

Nitrate content of commercial nonconcentrated beetroot juices can differ across the year.

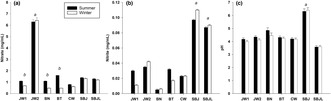

## 1 INTRODUCTION

Beetroot is one of the main dietary sources of inorganic nitrate $\left({\mathrm{NO}}_{3}^{-}\right)$ (Bailey et al., 2009), a natural ion that has been traditionally considered harmful due to the risk of formation of nitrosamines that can lead to cancer (Bryan et al., 2012; Zhang et al., 2019). As a consequence, the European Food Safety Authority (EFSA) established an acceptable daily intake (ADI) for ${\mathrm{NO}}_{3}^{-}$ of 3.7 mg/kg body mass/day that is still valid (EFSA et al., 2017). However, this view has substantially changed over the last decade due to new evidence suggesting that consumption of vegetables rich in ${\mathrm{NO}}_{3}^{-}$, which can exceed the ADI levels, is safe and it can enhance nitric oxide (NO) bioavailability (Hultström et al., 2015; Lundberg et al., 2018; Senefeld et al., 2020). Increased NO bioavailability due to ${\mathrm{NO}}_{3}^{-}$ consumption has been associated with enhanced exercise capacity especially in moderate‐trained individuals and some clinical populations (Senefeld et al., 2020; Shannon et al., 2022). Consequently, beetroot juice (BJ) is currently listed within category A (products with the most scientific evidence to enhance exercise performance) in the Sport Supplement Framework of the Australian Institute of Sport (Australian Institute of Sport [AIS], 2023).

The minimum amount of ${\mathrm{NO}}_{3}^{-}$ that can elicit improvements in exercise performance is about 5 mmol (310 mg) (Shannon et al., 2022); however, the content of ${\mathrm{NO}}_{3}^{-}$ in most commercial BJs is not indicated in the list of ingredients as it is not required by the law. This is important since the content of ${\mathrm{NO}}_{3}^{-}$ in vegetables can substantially change depending on several factors including environmental conditions, soil clay content, organic matter content, nitrogen fertilization, and type of beet (Gallardo & Coggan, 2019). Previous research in lettuce and spinach has shown large variations in the content of ${\mathrm{NO}}_{3}^{-}$ and nitrite $\left({\mathrm{NO}}_{2}^{-}\right)$ across different seasons being higher in the winter than in the summer (Ashworth & Bescos, 2017). Similar data in beets or BJ are missing, but it can be hypothesized that similar variations can also occur across the year.

Given the potential ergogenic effects of BJ, the popularity of this product is increasing among professional and recreational athletes with an average increase in the sales of beetroot of 8% per year since 2016 (Ysart et al., 1999). However, some people dislike the taste of this product (Grand View Research, 2022). One approach to make BJ more attractive is by mixing it with other juices such as lemon and apple juice. They can also act as natural preservatives due to their antioxidant compounds (e.g., ascorbic acid). However, the effect of adding lemon juice on the ${\mathrm{NO}}_{3}^{-}$ content of BJ has not been reported. This study investigated whether the addition of lemon juice to self‐made BJ affects the content of ${\mathrm{NO}}_{3}^{-}$ and ${\mathrm{NO}}_{2}^{-}$ and acidity (pH) levels.

Another important issue that has not been analyzed especially in commercial BJs is the stability of ${\mathrm{NO}}_{3}^{-}$. While small shots (70 mL) are easily consumed at once, large bottles (0.5–1 L) can last longer and the storage conditions can affect the ${\mathrm{NO}}_{3}^{-}$ content. Fresh BJ (from natural beets) may contain bacteria that can reduce ${\mathrm{NO}}_{3}^{-}$ into ${\mathrm{NO}}_{2}^{-}$ increasing the concentration of the second (Babateen et al., 2021). This is relevant because while inorganic ${\mathrm{NO}}_{3}^{-}$ is safe even at high doses, ${\mathrm{NO}}_{2}^{-}$ can cause serious harm at considerably lower levels. Thus, it is important to consider the content of ${\mathrm{NO}}_{2}^{-}$ in BJ as well. In this study, we investigated the effect of three different storage temperatures (room: 20°C; fridge: 4°C; and freezer: −20°C) on the content of ${\mathrm{NO}}_{3}^{-}$ and ${\mathrm{NO}}_{2}^{-}$ in commercial and self‐made BJ.

In summary, the main goals of this study were to: (1) analyze the content of ${\mathrm{NO}}_{3}^{-}$ and ${\mathrm{NO}}_{2}^{-}$ of commercial and self‐made BJs at different periods of the year; (2) analyze the effect of the storage temperature on the content of ${\mathrm{NO}}_{3}^{-}$ and ${\mathrm{NO}}_{2}^{-}$ in commercial and self‐made BJ; (3) investigate the effect of adding lemon into self‐made BJ on the ${\mathrm{NO}}_{3}^{-}$ and ${\mathrm{NO}}_{2}^{-}$ content and acidity (pH). According to this, the main hypotheses of this study were that: (1) ${\mathrm{NO}}_{3}^{-}$ content of commercial and self‐made BJ will differ across different periods of the year; (2) ${\mathrm{NO}}_{3}^{-}$ in commercial and self‐made BJ will be degraded more quickly at higher temperatures; (3) the addition of lemon juice into self‐made BJ will reduce ${\mathrm{NO}}_{3}^{-}$ degradation in self‐made BJ.

## 2 METHODS

We analyzed the ${\mathrm{NO}}_{3}^{-}$ and ${\mathrm{NO}}_{2}^{-}$ content in five commercial BJs that are commonly used by professional and recreational athletes and two self‐made BJs (Table 1). Commercial juices and raw beets were purchased in June 2021 and February 2022 and stored for less than 1 week at room temperature or under refrigeration as recommended by manufacturers before they were analyzed.

**TABLE 1:** Beetroot juices analyzed in this study.

Brand	Code	Product	Serving size (mL)	Claimed nitrate content (mg/serving)	Characteristics
Juices					
James White	JW1	Beet it organic juice	1000	800	90% organic beetroot juice + 10% organic apple juice
James White (300 mg)	JW2	Beet it sport	70	300	98% organic concentrated beetroot juice + 2% lemon juice
Biona	BN	Beetroot pressed juice	500	‐	Beetroot juice partially lacto‐fermented + lemon juice
Biotta	BT	Beetroot juice	500	‐	100% organic pressed beetroot juice lacto‐fermented
Cawston	CW	Brilliant beetroot juice	1000	‐	90% pressed beetroot juice + 10% pressed apple juice + vitamin C
Fresh beetroot juice	SBJ	Fresh beetroot juice 1	50	‐	100% pressed beetroot juice
Fresh beetroot juice + lemon juice	SBJL	Fresh beetroot juice	50	‐	95% pressed beetroot juice + 5% pressed lemon juice

### 2.1 Preparation of products

Self‐made beetroot juice (SBJ) was prepared using whole beets (*Beta vulgaris*) from a local supermarket (Plymouth, UK). Beetroot was washed with tap water, and then with ultrapure water (Purelab OptionQ). The outer skin and inedible parts were removed before being chopped into small pieces and weighed using an electronic scale (Precisa XB 3200C). Then, beetroot was juiced using an electric juicer machine (Waring 11JE65). Lemon was bought in a local supermarket and juiced using a fruit juicer. Then, 2.5 mL (5%) of lemon juice was mixed with 47.5 mL (95%) of fresh beetroot juice (SBJ), which is similar to the volume of lemon juice added into some commercial BJs analyzed in this study (Table 1). Commercial BJs were opened on the first day of analyses. All BJs were filtered using a Whatman® filter paper number 1 and centrifuged at 3500 rpm for 10 min to remove solid parts.

### 2.2 Analysis of nitrate $\left({\mathrm{NO}}_{3}^{-}\right)$ and nitrite $\left({\mathrm{NO}}_{2}^{-}\right)$

All beetroot samples were centrifuged at 13,000 rpm at 4°C for 10 min before analysis. The content of ${\mathrm{NO}}_{3}^{-}$ and ${\mathrm{NO}}_{2}^{-}$ of each product was analyzed using a dedicated high‐performance liquid chromatography analyzer (ENO‐30; Eicom USA) as previously described (Corleto et al., 2018). Briefly, ${\mathrm{NO}}_{3}^{-}$ and ${\mathrm{NO}}_{2}^{-}$ were separated on a reverse‐phase separation column packed with polystyrene polymer (NO‐PAK 4.6 × 50 mm, EICOM; Amuza, Inc.), and ${\mathrm{NO}}_{3}^{-}$ was reduced to ${\mathrm{NO}}_{2}^{-}$ in a reduction column packed with copper‐plated cadmium filings (NO‐RED EICOM; Amuza, Inc.). ${\mathrm{NO}}_{2}^{-}$ was mixed with a Griess reagent to form a purple azo dye in a reaction coil. The separation and reaction columns and the reaction coil were placed in a column oven set at 35°C. The absorbance of the color of the product dye at 540 nm was measured with a flow‐through spectrophotometer (NOD‐30; Eicom). The mobile phase (10% methanol, 0.15 M NaCl/NH_4_Cl, and 0.5 g/L 4Na‐EDTA) and reactor phase (10% methanol, 1.25% HCl containing 5 g/L of sulfanilamide with 0.25 g/L of *N*‐naphthylethylenediamine) were delivered at a flow rate of 0.33 mL/min and 0.10 mL/min, respectively. A standard curve was produced by injecting 10 μL of water with sodium ${\mathrm{NO}}_{3}^{-}$ (${\text{NaNO}}_{3}^{-}$/7631‐99‐4; Sigma Aldrich) and sodium ${\mathrm{NO}}_{2}^{-}$ (${\text{NaNO}}_{2}^{-}$/7632‐00‐0; Sigma Aldrich) at different concentrations (7.8, 15.6, 31.2, 62.5, 125, and 250 μM). Beetroot samples were diluted 1:200 using a carrier solution containing 10% methanol, 0.15 M NaCl/NH_4_Cl, and 0.5 g/L 4Na‐EDTA. Samples were analyzed (10 μL) in duplicate on the first day and single on third and seventh day given the small coefficient of variation of ${\mathrm{NO}}_{3}^{-}$ (2.1 ± 1.9%) and ${\mathrm{NO}}_{2}^{-}$ (4.8 ± 3.0%) analyses.

### 2.3 pH measurements

Measurements of pH were performed using a single‐electrode digital pH meter (Lutron Electronic Enterprise Co Ltd.; Model PH‐208) that was calibrated following the manufacturer's instructions prior to each use.

### 2.4 Storage temperature

The effect of different storage temperatures on the ${\mathrm{NO}}_{3}^{-}$ and ${\mathrm{NO}}_{2}^{-}$ content was only analyzed in the first batch (June 2021). Eppendorf (1.5 mL) and Falcon tubes (3 mL) were filled with each product and kept at three different temperatures (20°C, 4°C, and −20°C) to analyze ${\mathrm{NO}}_{3}^{-}$, ${\mathrm{NO}}_{2}^{-}$, and pH on the first (baseline), third, and seventh day using the same methods described above. All the tubes were wrapped with aluminum foil to preserve the samples from light oxidation. Samples at −20°C were thawed on the same day of the analysis. Then, all samples were centrifuged at 13,000 rpm at 4°C for 10 min before analysis was undertaken.

### 2.5 Statistical analyses

Data are presented as mean ± standard deviation. Differences in ${\mathrm{NO}}_{3}^{-}$ and ${\mathrm{NO}}_{2}^{-}$ content and pH between different BJs were compared using a one‐way analysis of variance. Post hoc analyses were performed using Tukey HSD. Data were analyzed using the statistical software SPSS (version 28). The level of significance was set at *p* < .05.

## 3 RESULTS

### 3.1 Juices

Raw beetroot (SBJ) in the summer (102 g) and winter (178 g) yielded 53 (52%) and 107 (60%) mL of juice, respectively.

### 3.2 ${\mathrm{NO}}_{3}^{-}$ and ${\mathrm{NO}}_{2}^{-}$ content in BJs in the summer and winter

**FIGURE 1:**
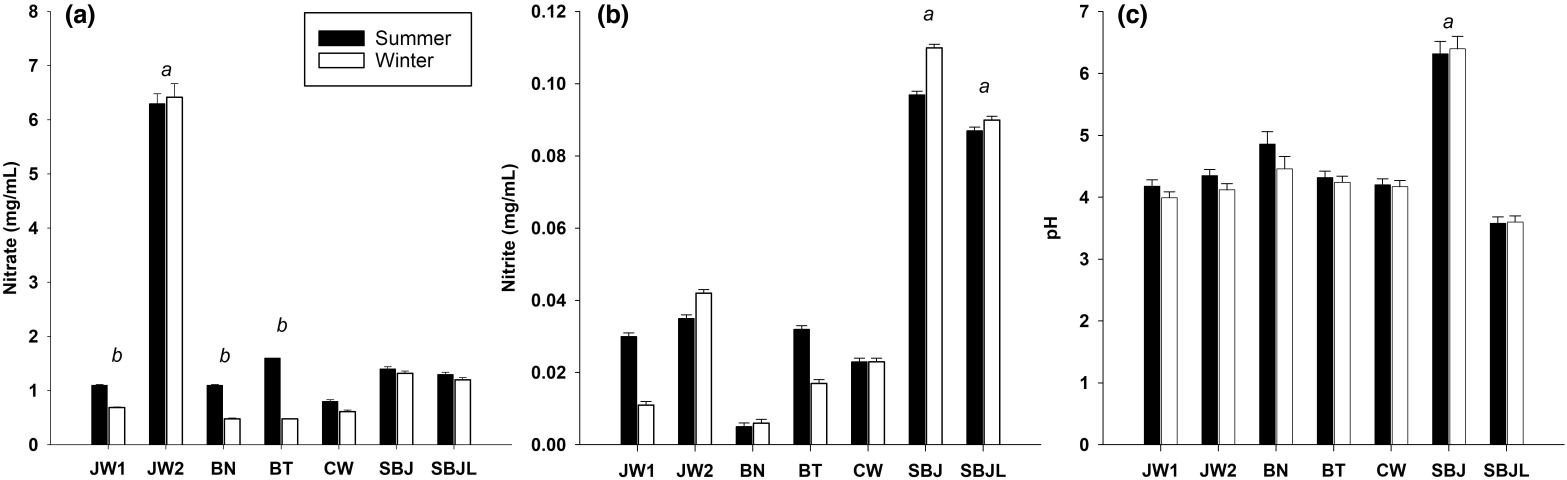
Content of nitrate $\left({\mathrm{NO}}_{3}^{-}\right)$ (A), nitrite $\left({\mathrm{NO}}_{2}^{-}\right)$ (B), and pH (C) in commercial and self‐made beetroot juices in two different periods of the year. (*a* represents statistical differences between beetroot juices; *b* represents statistical differences between beetroot juice batches in the summer and winter.

As expected, concentrated BJ (JW2) had the highest content of ${\mathrm{NO}}_{3}^{-}$ (6.3 ± 0.2 mg/mL; *p* = .001) compared to nonconcentrated commercial (JW1: 1.1 ± 0.2 mg/mL; BN: 1.1 ± 0.1 mg/mL; BT: 1.6 ± 0.2 mg/mL; CW: 0.8 ± 0.1 mg/mL) and self‐made juices (SBJ: 1.4 ± 0.2 mg/mL; SBJL: 1.3 ± 0.2 mg/mL) (Figure 1A). The content of ${\mathrm{NO}}_{3}^{-}$ of concentrated BJ (JW2) was similar in the summer (6.3 ± 0.2 mg/mL) and winter (6.4 ± 0.2 mg/mL; *p* > .05). Nonconcentrated BJs (JW1, BN, BT, CW, and SBJ), had on average 34 ± 20% more ${\mathrm{NO}}_{3}^{-}$ in the summer (1.2 ± 0.3 mg/mL) than in the winter (0.8 ± 0.3 mg/mL; *p* = .075) (Figure 1A). These differences were more pronounced in JW1, BN, and BT juices (from 0.7 ± 0.1, 0.5 ± 0.1, and 0.8 ± 0.1 mg/mL in the winter to 1.1 ± 0.1, 1.1 ± 0.1, and 1.6 mg/mL in the summer; *p* < .001) than in CW and SBJ (from 0.6 ± 0.1 and 1.3 ± 0.2 mg/mL in the winter to 0.8 ± 0.1 and 1.4 ± 0.2 mg/mL in the summer; *p* > .05) (Figure 1A).

**FIGURE 2:**
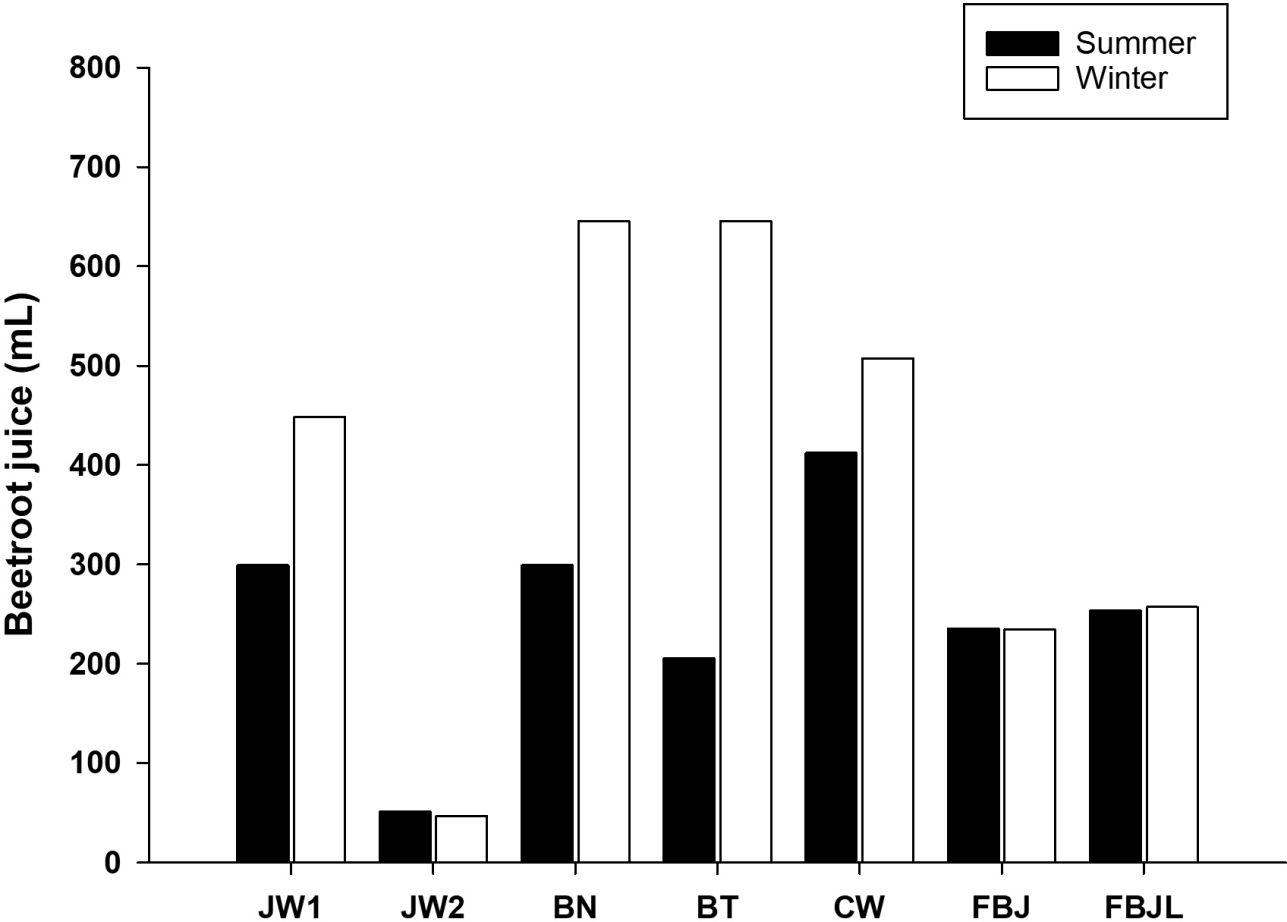
Estimated amount of beetroot juice required to achieve 5 mmol of nitrate $\left({\mathrm{NO}}_{3}^{-}\right)$ from commercial and self‐made beetroot juices in the summer and winter.

Using the ${\mathrm{NO}}_{3}^{-}$ results from each product, we calculated the amount of BJ that was needed to achieve the minimum dose of ${\mathrm{NO}}_{3}^{-}$ to enhance exercise performance (5 mmol of ${\mathrm{NO}}_{3}^{-}$ = 310 mg) (Figure 2). With the exception of concentrated (JW2) and self‐made juice (SBJ), an average of 258 ± 162 mL more BJ from the winter batches of commercial BJs was needed to achieve such amount compared to the summer batches.

The content of ${\mathrm{NO}}_{2}^{-}$ is shown in Figure 1B. SBJ (0.097 ± 0.01 mg/mL) and SBJL (0.090 ± 0.01 mg/mL) had the highest content of ${\mathrm{NO}}_{2}^{-}$ compared to the commercial BJs (JW1: 0.030 ± 0.01 mg/mL; JW2: 0.035 ± 0.01 mg/mL; BN: <0.01 ± 0.01 mg/mL; BT: 0.032 ± 0.01 mg/mL; CW: 0.023 ± 0.01 mg/mL; *p* = .001) (Figure 1B). The content of ${\mathrm{NO}}_{2}^{-}$ was slightly lower in JW1 and BT juices in the winter (JW1: 0.011 ± 0.01 mg/mL; BT: 0.017 ± 0.01 mg/mL) than in the summer (JW1: 0.030 ± 0.01 mg/mL; BT: 0.032 ± 0.01 mg/mL; *p* = .110), while JW2 had slightly higher content of ${\mathrm{NO}}_{2}^{-}$ in the winter (0.110 ± 0.01 mg/mL) than in the summer (0.097 ± 0.01 mg/mL; *p* = .101).

### 3.3 pH of BJ

Results of pH are shown in Figure 1C. SBJ had the highest pH (6.3 ± 0.1) compared to commercial juices (mean pH from all the commercial BJs = 4.5 ± 0.3; *p* = .001) and SBJL (3.6 ± 0.1; *p* = .001) (Figure 1C). Overall, the average pH of commercial juices (JW1, JW2, BN, BT, and CW) was slightly lower in the winter (4.2 ± 0.2) than in the summer (4.5 ± 0.3; *p* = .239).

### 3.4 Effect of storage temperature on ${\mathrm{NO}}_{3}^{-}$, nitrite, and pH

**FIGURE 3:**
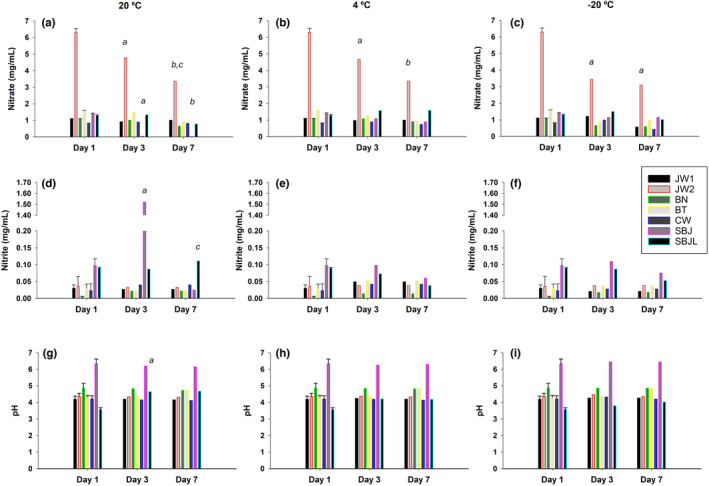
Effect of storage temperature on the content of nitrate $\left({\mathrm{NO}}_{3}^{-}\right)$ (A–C), nitrite $\left({\mathrm{NO}}_{2}^{-}\right)$ (D–F), and pH (G–I) of beetroot juice at baseline (day 1) and 3 and 7 days after opening the package (commercial beetroot juice) or after preparing self‐made beetroot juice. (*a* represents statistical differences between day 1 and 3; *b* represents statistical differences between day 1 and 7; *c* represents statistical differences between day 3 and 7).

The content of ${\mathrm{NO}}_{3}^{-}$ in juices stored at 20, 4, and −20°C for 1, 3, and 7 days during the summer is shown in Figure 3.

A reduction of 24% (from 6.3 ± 0.2 to 4.8 ± 0.2 mg/mL; *p* < .001) and 46% (from 6.3 ± 0.2 to 3.4 ± 0.2 mg/mL, *p* < .001) in ${\mathrm{NO}}_{3}^{-}$ was observed when concentrated BJ (JW2) was kept at 20°C for 3 and 7 days, respectively (Figure 3A). A similar effect was observed when JW2 was kept for 3 days at 4°C (from 6.3 ± 0.2 to 4.7 ± 0.2 mg/mL; *p* < .001) and −20°C (from 6.3 ± 0.2 to 3.4 ± 0.2 mg/mL; *p* < .001) (Figure 3B).

${\mathrm{NO}}_{3}^{-}$ was degraded in SBJ after 3 days at 20°C (from 1.4 ± 0.1 to 0.04 ± 0.01 mg/mL; *p* < .001) (Figure 3A). This reduction was attenuated by 78% (from 1.4 ± 0.1 to 1.1 ± 0.1 mg/mL) and 82% (from 1.4 ± 0.1 to 1.2 ± 0.1 mg/mL) when it was kept at 4 and −20°C for 3 days, respectively (Figure 3B,C).

The addition of 5% lemon juice was also effective to fully attenuate the reduction of ${\mathrm{NO}}_{3}^{-}$ in SBJ for 3 days (from 1.3 ± 0.1 to 1.3 ± 0.1 mg/mL) at 20°C (Figure 3A). Furthermore, the addition of lemon juice was useful to preserve 62% of ${\mathrm{NO}}_{3}^{-}$ in SBJ (from 1.3 ± 0.1 to 0.8 ± 0.1 mg/mL) when it was kept at 20°C for 7 days (Figure 3A).

Regarding the ${\mathrm{NO}}_{2}^{-}$ content, an abrupt increase was observed in SBJ on day 3 at 20°C (from 0.097 ± 0.01 to 1.5 ± 0.2 mg/mL; *p* < .001) (Figure 3D). This effect was inhibited when SBJ was stored at 4°C for 3 (from 0.097 ± 0.01 to 0.01 ± 0.001 mg/mL) and 7 days (from 0.097 ± 0.01 to 0.01 ± 0.001 mg/mL) and when it was stored at −20°C for the same duration (3 days: from 0.01 ± 0.001 to 0.01 ± 0.001 mg/mL; 7 days: from 0.01 ± 0.001 to 0.01 ± 0.001 mg/mL) (Figure 3E,F). Furthermore, the addition of lemon juice to self‐made juice (SBJL) was effective to inhibit the increase in ${\mathrm{NO}}_{2}^{-}$ when it was kept at 20°C for 3 (from 0.097 ± 0.01 to 0.087 ± 0.01 mg/mL) and 7 days (from 0.090 ± 0.01 to 0.11 ± 0.01 mg/mL), respectively (Figure 3D).

The pH of all juices, except in SBJL, remained relatively stable on days 3 and 7 at 20, 4, and −20°C (Figure 3G–I). The pH of SBJL increased from day 1 to 3 when it was kept at 20°C (from 3.6 ± 0.1 to 4.7 ± 0.1; *p* < .001).

## 4 DISCUSSION

The main finding of this study was that the content of ${\mathrm{NO}}_{3}^{-}$ in nonconcentrated commercial BJs was on average 34 ± 20% lower in the winter than in the summer. Differences in the content of ${\mathrm{NO}}_{3}^{-}$ in concentrated commercial (JW2) (1.9 ± 0.7%) and SBJs (SBJ, SBJL) (5.7 ± 2.1%) were smaller.

${\mathrm{NO}}_{3}^{-}$ is the main form of nitrogen used by crops to synthesize amino acids. They absorb ${\mathrm{NO}}_{3}^{-}$ from the soil via transporter proteins in the root cell membrane (Lundberg et al., 2011). Thus, the amount of ${\mathrm{NO}}_{3}^{-}$ in vegetables depends on the level of this ion in the soil, which can substantially differ across the year. For example, in the UK, it has been reported that the soil is poorer in ${\mathrm{NO}}_{3}^{-}$ in the winter because wet conditions (rainfalls) can wash out ${\mathrm{NO}}_{3}^{-}$ into the groundwater, a phenomenon known as ${\mathrm{NO}}_{3}^{-}$ leaching (Thomas et al., 2019). For this reason, it is feasible to use additional fertilizer (nitrogen) in autumn and winter in some vulnerable areas to improve the crops yield (Thomas et al., 2019). Four of the commercial BJs (JW1, JW2, BN, and BT) analyzed in this study indicated that beetroot used was organic so nitrogen fertilizers were not supposed to be used during the growth of the crops. Interestingly, all of them, except the concentrated juice (JW2), had lower content of ${\mathrm{NO}}_{3}^{-}$ when they were bought and analyzed in the winter, which may suggest that beetroot were grown over the summer. However, this information was not provided by the commercial companies. Light conditions, use of organic matter (animal manure), and storage conditions are also important factors to take into account as they can affect the content of ${\mathrm{NO}}_{3}^{-}$ in vegetables (Dechorgnat et al., 2010; Gov.UK, 2022; Santamaria, 2006). Commercial companies can obtain beetroot from different locations and areas given the large amount of product needed to constantly supply the market, which can modify the content of ${\mathrm{NO}}_{3}^{-}$ in the final product. Furthermore, there is no regulation about labeling the content of ${\mathrm{NO}}_{3}^{-}$ in commercial BJ, its origin, or when crops were harvested. This is relevant given the potential physiological implications of ${\mathrm{NO}}_{3}^{-}$ and the variations in the content of this ion observed in this study in some commercial products. Although individuals can always choose to consume larger‐than‐recommended amounts, potential disadvantages to doing so include increased cost, greater volume to ingest, higher intake of oxalate, and potential side effects.

Only two of the commercial juices analyzed in this study (JW1 and JW2) reported an estimated value of ${\mathrm{NO}}_{3}^{-}$ in the serving size (Table 1). The first juice (JW1) claimed that the ${\mathrm{NO}}_{3}^{-}$ content was on average 800 mg per liter (0.8 mg/mL). Compared to this, we found that the ${\mathrm{NO}}_{3}^{-}$ content of this product was 38% higher in the summer batch (June 2020) (1.1 mg/mL) and 14% lower in the winter batch (February 2021) (0.69 mg/mL). On the other hand, the ${\mathrm{NO}}_{3}^{-}$ content from a concentrated product from the same commercial brand (JW2) was 47% and 50% higher in summer and winter batches compared to the claimed ${\mathrm{NO}}_{3}^{-}$ content. Our results are in agreement with a previous study indicating that the ${\mathrm{NO}}_{3}^{-}$ content of the same BJ was 23% higher than the claimed ${\mathrm{NO}}_{3}^{-}$ content (Weightman et al., 2012). However, they did not compare the content of ${\mathrm{NO}}_{3}^{-}$ of the same product across different periods of the year. Furthermore, both studies showed that commercial concentrated beetroot shots (JW2) had nearly five times more ${\mathrm{NO}}_{3}^{-}$ than commercial nonconcentrated and fresh BJ. Concentrated beetroot shots appeared in the market a decade ago to provide the minimal dose of inorganic ${\mathrm{NO}}_{3}^{-}$ (5 mmol = 310 mg/serving) that has been suggested to enhance exercise capacity in a small volume (Santamaria et al., 1999). Although the method to concentrate BJ is not reported on the label, this process is usually performed by removing part of the water from the juice (Jones et al., 2018).

Regarding the effect of temperature storage on the content of ${\mathrm{NO}}_{3}^{-}$ and ${\mathrm{NO}}_{2}^{-}$, rapid degradation of ${\mathrm{NO}}_{3}^{-}$ occurred in SBJ when it was kept at 20°C for 3 days, but this reaction was attenuated by storing it at low temperatures and by adding lemon juice (SBJL), a natural source of ascorbic acid. This is in agreement with our hypothesis, suggesting that low temperatures and the addition of lemon juice can help to attenuate ${\mathrm{NO}}_{3}^{-}$ degradation in BJ. Ascorbic acid is widely used in the food industry for its antioxidant and stabilizing properties (Bazaria & Kumar, 2016). Two of the commercial juices analyzed in this study (JW2 and BN) contained lemon juice, two more apple juice (JW1 and CW), and another one (CW) was fortified with ascorbic acid. Despite the addition of lemon juice into concentrated BJ (JW2), we found a rapid reduction in the content of ${\mathrm{NO}}_{3}^{-}$ occurred over days 3 and 7 that was not attenuated at low temperatures. According to this, rapid consumption of concentrated BJ is recommended to enhance ${\mathrm{NO}}_{3}^{-}$ intake. This is in agreement with the recommendations from the commercial companies indicating to keep the juice refrigerated and consume it within 3–7 days once opened. The addition of lemon and apple juices can also help to enhance the organoleptic characteristics of BJ for some people who dislike the taste of beetroot (Grand View Research, 2022).

The content of ${\mathrm{NO}}_{2}^{-}$ was very low (<0.1 mg/mL) in all the juices at baseline; however, a rapid increase was observed in SBJ on day 3 at 20°C. This could happen due to the activity of ${\mathrm{NO}}_{3}^{-}$ reductase enzymes or microorganisms present in beetroot as the decrease of ${\mathrm{NO}}_{3}^{-}$ was accompanied by the increase of ${\mathrm{NO}}_{2}^{-}$. From a safety point of view, the levels of ${\mathrm{NO}}_{2}^{-}$ achieved on day 3 were quite low to cause harm in healthy individuals as doses above 100 mg/kg of body mass are required to produce serious side effects in humans (Liao & Seib, 1988). According to our results, the consumption of over 4 L of BJ rich in ${\mathrm{NO}}_{2}^{-}$ over a relatively short period of time may be needed to reach this quantity of ${\mathrm{NO}}_{2}^{-}$. However, a word of caution is needed about BJ overload among athletes thinking ‘the more the better’.

The pH of commercial BJs was more acidic than self‐made juice (SBJ), which can be related to lacto‐fermentation and addition of ascorbic acid in commercial juices. Two commercial juices of this study were lacto‐fermented (BN and BT), which consists of the addition of lactic acid bacteria consuming sugars to produce acid compounds and carbon dioxide by fermentation (Klewicka et al., 2015). Three commercial juices also contained lemon juice (JW2 and BN) or vitamin C as an additive (CW). The addition of lemon juice to SBJ (SBJL) is a useful approach to maintain the content of ${\mathrm{NO}}_{3}^{-}$ as we demonstrated in this study. On the other hand, further research is needed to investigate whether lacto‐fermentation and/or addition of other juices can modify ${\mathrm{NO}}_{3}^{-}$ bioavailability.

This study had some limitations that are worth discussing. First, it was based on a Master's thesis that was performed during Covid‐19 pandemic when students had to deal with laboratory restrictions. Bottles of five of the most consumed brands of BJ in the UK were analyzed in the summer (June) and winter (February) seasons. The batch code of each product was not recorded, but we believe that juices belonged to different batches given the time gap (8 months) between the purchase and analyses of them. Despite this limitation, our results are still interesting, indicating that the content of ${\mathrm{NO}}_{3}^{-}$ can substantially differ especially in commercial nonconcentrated BJs. We also had limitations to increase the sample size of different BJs given the duration of each chromatogram (10 min) to analyze ${\mathrm{NO}}_{3}^{-}$ and ${\mathrm{NO}}_{2}^{-}$. We could analyze a maximum of 42 samples in a day. In the winter batches (February 2021), only baseline analyses of ${\mathrm{NO}}_{3}^{-}$, ${\mathrm{NO}}_{2}^{-}$, and pH were performed due to time constraints. All the analyses (${\mathrm{NO}}_{3}^{-}$, ${\mathrm{NO}}_{2}^{-}$, and pH) were performed in duplicate during the first day (7 samples × 3 different temperatures) to ensure the reproducibility of the results. Regarding the effect of storage temperature, only 1.5 and 3 mL of each BJ were taken and stored at the respective temperature prior to testing, which may not represent what would happen in larger volumes (e.g., 500 mL) of juice.

In summary, this study showed that the ${\mathrm{NO}}_{3}^{-}$ content of commercial BJs can substantially differ across different batches. Reduction of the content of ${\mathrm{NO}}_{3}^{-}$ in concentrated commercial BJ and fresh BJ occurs quickly at room temperature (20°C). Furthermore, it is possible to obtain similar quantities of ${\mathrm{NO}}_{3}^{-}$ from self‐made BJ compared to nonconcentrated commercial BJs, but it must be kept at low temperatures (4 and −20°C) and/or mixed with lemon juice to avoid ${\mathrm{NO}}_{3}^{-}$ degradation. These findings are relevant to individuals (e.g., nutritionists, athletes, coaches, etc.) and researchers interested in the physiological effects of BJ supplementation. Indeed, given the possible variation in the ${\mathrm{NO}}_{3}^{-}$ content of BJ, scientists looking at the physiological effect of dietary ${\mathrm{NO}}_{3}^{-}$ in BJ should measure the content of ${\mathrm{NO}}_{3}^{-}$ in the supplement.

## AUTHOR CONTRIBUTIONS

Raul Bescos and Mark L. Rollason contributed to project conception. Raul Bescos, Mark L. Rollason, Tanisha S. Davies, and Patricia Casas‐Agustench analyzed the data. Raul Bescos, Mark L. Rollason, Tanisha S. Davies, and Patricia Casas‐Agustench authors contributed to data interpretation. Raul Bescos and Patricia Casas‐Agustench drafted the initial paper. All authors revised and approved the final manuscript.

## CONFLICT OF INTEREST STATEMENT

The authors declare no conflicts of interest.

## Data Availability

Data will be available on request from the authors.
